# Allogeneic hematopoietic stem cell transplantation in adult acute lymphoblastic leukemia: potential benefit of medium-dose etoposide conditioning

**DOI:** 10.1186/s40164-015-0015-0

**Published:** 2015-07-16

**Authors:** Masahiro Imamura, Akio Shigematsu

**Affiliations:** Department of Hematology, Sapporo Hokuyu Hospital, Higashi-Sapporo 6-6-5-1, 003-0006 Shiroishi-ku, Sapporo Japan

**Keywords:** Acute lymphoblastic leukemia, Hematopoietic stem cell transplantation, Myeloablative conditioning regimen, Reduced-intensity conditioning regimen, Cyclophosphamide, Etoposide, Total body irradiation, Haploidentical HSCT, Philadelphia chromosome, Tyrosine kinase inhibitor

## Abstract

The outcomes of adult acute lymphoblastic leukemia (ALL) patients with chemotherapy or autologous hematopoietic stem cell transplantation (HSCT) are unsatisfactory. Therefore, allogeneic (allo) HSCT has been applied to those patients in first complete remission (CR1), and has shown a long-term survival rate of approximately 50 %. In terms of myeloablative conditioning (MAC) regimen, higher dose of cyclophosphamide (CY) and total body irradiation (TBI) (the standard CY + TBI) has been generally applied to allo HSCT. Other MAC regimens such as busulfan-based or etoposide-based regimens have also been used. Among those, medium-dose etoposide (ETP) in addition to the standard CY + TBI conditioning regimen appears to be promising for allo HSCT in adult ALL when transplanted in ALL patients aged under 50 years in CR1 and also in CR2, showing an excellent outcome without increasing relapse or transplant-related mortality (TRM) rates. By contrast, reduced-intensity conditioning (RIC) regimens have also been applied to adult ALL patients and favorable outcomes have been obtained; however, relapse and TRM rates remain high. Therefore, an allo HSCT conditioning regimen which deserves further study for adult ALL patients aged under 50 years in CR1 and CR2 appears to be medium-dose ETP + CY + TBI and RIC is suitable for patients aged over 50 years or for younger patients with comorbid conditions. On the contrary, new therapeutic strategies for adult ALL patients are increasingly utilized with better outcomes; namely, various tyrosine kinase inhibitors for Philadelphia chromosome (Ph)-positive ALL, human leukocyte antigen-haploidentical HSCT, and pediatric-inspired regimens for Ph-negative ALL. Therefore, the optimal treatment modality should be selected considering patient’s age, Ph-positivity, donor availability, risk classification, efficacy, and safety.

## Introduction

Adult acute lymphoblastic leukemia (ALL) is a type of intractable hematological malignancies, showing a long-term survival rate of approximately 30 % with a high incidence of relapse when treated with intensive chemotherapies [30, 53, 70, 74]. Allogeneic (allo) hematopoietic stem cell transplantation (HSCT) from a human leukocyte antigen (HLA)-matched donor has been applied to ALL patients, and showed a superior response to chemotherapy and autologous (auto) HSCT [23, 70], although there is some controversy regarding the efficacy of allo HSCT for ALL patients, especially for standard-risk patients [58, 70]. Although it was suggested that some high-risk patients should be transplanted in first complete remission (CR1) [58], standard-risk patients in CR1 had a more beneficial effect by allo HSCT [23]. A similar result was shown in another report that compared the efficacy of allo HSCT versus auto HSCT for standard-risk adult ALL patients in CR1, showing a 5-year (Y) disease free survival (DFS) rate of 69 % versus 45 % and a 5-Y relapse rate of 14 % versus 52 %, respectively [14]. Furthermore, a 5-Y estimated non-relapse mortality (NRM) rate was 16 % versus 2 %. These outcomes of allo HSCT for adult ALL patients are unsatisfactory. Generally, long-term survival rates of 40-60 % are obtained when both child and adult high-risk patients in CR1 or second complete remission (CR2) received allo HSCT by conventional myeloablative conditioning (MAC) regimens [27, 34, 35, 42, 70]. The outcome decreased depending on the remission status at transplantation; namely, 40-60 % in CR1, 20-40 % in CR2, 10-20 % in ≧ third complete remission (CR3), and approximately 10 % in non-CR [5, 18, 26, 34, 35, 42, 47]. A standard conventional MAC regimen for allo HSCT in patients with ALL is 120 mg/kg cyclophosphamide (CY) plus 12 to 13.2 gray (Gy) fractionated total body irradiation (TBI). With this regimen, a 3-Y overall survival (OS) or DFS rate of approximately 50 % was obtained when transplanted in CR1 [58, 70].

By contrast, reduced-intensity conditioning (RIC) regimens have been applied to older patients and younger patients with comorbid conditions. Fludarabine (Flu)-based regimens are often utilized, showing an approximately 50 % OS rate; however, the frequencies of relapse and complications following HSCT are relatively high. Similar outcomes have been observed between RIC and MAC regimens in the Philadelphia chromosome (Ph)^+^ ALL patients who were minimal residual disease (MRD)-negative when transplanted in CR1 [2]. Therefore, RIC regimens are appropriate for patients aged ≧50 years or those <50 years with serious comorbid conditions and who are MRD-negative.

Recently, excellent outcomes have been obtained with pediatric-inspired regimens for adult Ph^−^ ALL patients [15, 16] and also with HLA-haploidentical (haplo) HSCT for ALL patients lacking an HLA-matched donor [1, 12, 40, 51, 54]. Furthermore, in Ph^+^ ALL patients, the introduction of tyrosine kinase inhibitors (TKIs) has revolutionized the treatment paradigm of Ph^+^ leukemia patients [28, 38, 39, 50, 74], although these strategies have some limitations. Therefore, it should be determined which type of treatment is optimal for each patient, considering patient’s age, Ph-positivity, donor availability, risk, efficacy, and safety.

### MAC regimens other than the CY + TBI regimen for adult ALL patients

MAC regimens other than the CY + TBI regimen, such as 60 mg/kg etoposide (ETP) + 12 - 13.2 Gy TBI or 16 mg/kg busulfan (BU) + 120 mg/kg CY, were also utilized in allo HSCT for hematological malignancies, including ALL (Table 1) [6, 7, 25, 37, 49, 57, 59–61, 64, 66]. A single dose or a one-day dose of ETP was generally used in ETP-based regimens. The outcome of BU (oral) + CY regimen was not superior to that of CY + TBI regimen. The outcome of ETP + TBI regimen was similar to that of CY + TBI regimen, although ETP as a substitute for CY is less immunosuppressive, resulting in more frequent engraftment failure [57]. An early phase I/II study for patients with advanced hematological malignancies of 33 patients with acute leukemia and 13 patients with ALL who were not in CR1, and 14 patients with other advanced hematological malignancies showed that a single dose of 60 mg/kg ETP was the maximal tolerated dose, as determined by relapse and survival rates (15 % and 54 %, respectively) with the dose ranging from 25 to 70 mg/kg [2].

**Table 1:** Myeloablative conditioning regimens in allogeneic hematopoietic stem cell transplantation for hematological malignancies, especially for acute lymphoblastic leukemia

Regimen	No. of ALL	Mean age (range)	Donor	Stem cell source	Disease status at HSCT	Survival rate	Relapse rate	TRM/NRM	aGVHD	cGVHD	Remarks	Reference
ETP 60 mg/kg × 1 + TBI 1.2 Gy × 11	13	16 (6–36)	MRD	BM	CR2, IF, or relapse	54 % (≧100 d)	15 %	31 %	23 %	ND	It was not clear whether ETP 60 mg/kg was better than ETP 30 mg/kg according to a phase I/II (dose-finding) study using 25 to 70 mg/kg of ETP.	Blume KG, et al. Blood 1987;69:1015.
ETP 50 to 70 mg/kg × 1 + TBI 2 Gy × 6	17	19 (4–38)	MRD	BM	Non-CR1, IF, or relapse except 1	65 % (≧182 d)	6 %	35 %	85 %	50 %	Anti-leukemic effect was observed in a phase I/II study with ETP + BI, but the rejection rate was high. Thus, the immunosuppressive effect was worse than CY + TBI. A dose of more than 60 mg/kg of ETP was too toxic.	Schmitz N, et al. Blood 1988;72: 1567.
ETP 36 mg/kg or 52 mg/kg × 1 + CY 67 mg/kg or 103 mg/kg × 1 + TBI 2 Gy × 6	7	15 (6–35)	MRD	BM	Relapse	0 % (≧899 d)		ND	ND	ND	ETP (36 mg/kg) + CY (67 mg/kg) + TBI was well tolerated for allogeneic HSCT (phase I study).	Petersen FB, et al. Bone Marrow Transplant. 1992; 10:83.
ETP 60 mg/kg × 1 + TBI 1.2 Gy × 11	25/122	2–48	MRD	BM	CR2: 8	3-Y DFS: 63 %	8 %	4 %	ND	ND	ETP + TBI appeared to be better for a good-risk group than for a poor-risk one according to a randomized controlled study. Hepatic toxicity and severe mucositis were marked with ETP of 60 mg/kg. The age range for the patients with ALL was shown, but the mean was unclear.	Blume KG, et al. Blood 1993;81: 2187.
					CR3, Non-CR: 17	12 %	40 %	12 %	ND	ND		
BU 1 mg/kg × 16 + CY 60 mg/kg × 2	23/122	5–48			CR2: 6	4 %	17 %	4 %	ND	ND		
					CR3, Non-CR: 17	17 %	34 %	22 %	ND	ND		
ETP 60 mg/kg × 1 + TBI 1.2 Gy × 11	34	27 (1–45)	MRD	BM	CR1	3-Y DFS: 64 %	12 %	ND	18 % (gr.≧II)	38 %	A relatively good outcome was observed in a phase II study, but relapse and TRM rates were high. Thirty of the 34 patients were high-risk.	Snyder DS, et al. Blood 1993;82: 2920.
						High-risk						
						<20 y 3-Y DFS : 100 %	0 %					
						≧20 y 3-Y DFS: 54 %	17 %					
ETP 30–60 mg/kg × 1 + CY 60–200 mg/kg × 2 + TBI 1.2Gy × 11 (1.5 Gy × 8)	20/44	18 (1–54)	MRD	BM	ND	Early death: standard-risk 2/18 high-risk 2/26	ND	ND	ND	ND	Fourty-four patients with hematological malignancies were analyzed in a retrospective study. Among them, 20 patients were ALL. Sixty to 65 mg/kg of ETP resulted in a fatal toxicity, but 30 to 50 mg/kg did not. Fifty mg/kg of ETP was considered to be the maximally tolerated dose.	Spitzer TR, et al. Int. J. Radiat. Oncol. Biol. Phys. 1994;29:39
ETP 25–60 mg/kg × 2	10/32	18 (3–49)	MRD	BM	ND	Early death: standard-risk 1/12 high-risk 5/20	ND					
BU 1 mg/kg × 12–16												
Standard		20 (1–39)	MRD	BM	CR2: 20	7-Y OS: 52 %	34 %	25 %	48 % (gr.≧II)	41 %	A retrospective study suggested that high-dose conditioning regimens did not improve the outcome of patients transplanted for high-risk leukemia.	Mengarelli A, et al. Haematologica 2002;87:52.
TBI 2 Gy × 6 + CY 120 mg/kg × 2	7/38				CR3: 9							
					1st Rel.: 5							
					Adv.: 4							
BU 16 mg/kg × 4 + CY 120 mg/kg × 2	24/38											
BU 16 mg/kg × 4 + CY 120 mg/kg × 4	7/38											
Alternative		23 (3–44)	MRD	BM: 60	CR2: 47	7-Y OS: 25 %	58 %	32 %	47 % (gr.≧II)	44 %		
ETP 60 mg/kg × 1 + TBI 2 Gy × 6 TBI 2 Gy × 6 + CY 120 mg/kg × 2	43/66			PB: 6	CR3: 9							
					1st Rel.: 6							
					Adv.: 13							
BU 16 mg/kg × 4 + CY 120 mg/kg × 2 +IDA 42 mg/m^2^ × 2												
BU-CY + VP 20 mg/kg × 1												
BU-CY + Ara-C 2 g/m^2^ × 4												
ETP 40 mg/kg × 1 + CY 60 mg/kg × 2 + TBI 2 Gy × 6	39	34 (15–52)	MRD: 35	BM	CR1	6-Y OS: 41 %	10 %	15 %	ND	ND	Autologous HSCT by ETP + CY + TBI regimen showed 41 % of 6-Y OS in a prospective study, whereas allogeneic BMT with the same regimen showed a 6-Y OS of 75 %. This result suggested a possibility of GVL effect. ETP was administered in 4 consecutive infusions of 10 mg/kg lasting 2 hours each.	Hunault M, et al. Blood 2004;104:3028.
			MUD: 4			(<50 years old: 75 %)						
ETP 15 mg/kg × 2 + CY 60 mg/kg × 2 + TBI 2 Gy × 6	37	26 (15–58)	MRD: 13	BM: 3	CR1: 28	3-Y OS: 89 %	8 %	5 %	78 % (gr.≧II: 41 %)	55 % (ext.: 36 %)	Excellent outcome was observed in addition to low relapse and TRM rates in a retrospective study.	Shigematsu A, et al. Biol. Blood Marrow Transplant. 2008;14:568.
			MUD: 18	PB: 4	CR2: 7							
			MMRD: 2	CB: 1	Non-CR: 2							
			MMUD: 4									
ETP 15 mg/kg × 2 + CY 60 mg/kg × 2 + TBI 2 Gy × 6 or 3 Gy × 4	35	28 (15–58)	MRD:16	BM: 29	CR1: 28	5-Y OS:82 %	14 %	3 %	71 % (gr.≧II: 37 %)	46 % (ext.: 30 %)	A retrospective analysis in Japan showed ETP + CY + TBI was associated with lower relapse and NRM rates, resulting in better survival than that with CY + TBI.	Shigematsu A, et al. Int. J. Hematol. 2011;94:463.
			MUD: 11	PB: 6	CR2:7							
			MMUD: 6									
			Unknown: 2									
CY 60 mg/kg × 2 + TBI 2 Gy × 6 or 3 Gy × 4	494	34 (15–59)	MRD: 235	BM: 405	CR1: 414	5-Y OS: 55 %	29 %	16 %	62 % (gr.≧II: 37 %)	45 % (ext.: 27 %)		
			MUD: 180	PB: 89	CR2: 80							
			MMRD: 1									
			MMUD: 70									
			Unknown: 2									
ETP 15 mg/kg × 2 + CY 60 mg/kg × 2 + TBI 2 Gy × 6	50	34 (17–49)	MRD: 26	BM: 40	CR1: 47	1-Y OS: 80 %	10 %	14 %	66 % (gr.≧II: 58 %; gr.≧III: 12 %)	56 % (ext.: 38 %)	A prospective multi-center phase II study in Japan confirmed the excellent outcome of ETP + CY + TBI for adult ALL patients.	Shimemastu A, et al. Transplant. Direct. 2015;1:1
			MUD: 24	PB: 10	CR2: 3	2-Y OS: 67 %						
						1-Y EFS: 76 %						
						2-Y EFS: 65 %						

*No*. the number of patients, *ALL* acute lymphoblastic leukemia, *HSCT* hematopoietic stem cell transplantation, *TRM* transplant-related mortality, *NRM* non-relapse mortality, *aGVHD* acute graft-versus-host disease, *cGVHD* chronic graft-versus-host disease, *ETP* etoposide, *TBI* total body irradiation, *MRD* matched related donor, *BM* bone marrow, *CR* complete remission, *IF* involved field, *d* day, *ND* not determined, *CY* cyclophosphamide, *Auto* autologous, *BU* busulfan, *Y* year, *DFS* disease-free survival, *MUD* matched unrelated donor, *OS* overall survival, *GVL* graft versus-leukemia, *MMRD* mismatched related donor, *MMUD* mismatched unrelated donor, *CB* cord blood, *PB* peripheral blood, *ext.* extensive type of cGVHD, *gr.* grade

### Comparison of the medium-dose ETP + CY + TBI regimen in the Hokkaido University Hospital with that in French group

We tried to examine whether more intensified conditioning regimens of adding 10–25 mg/kg/day for 2 days of ETP to the CY (60 mg/kg × 2) + TBI (2 Gy × 6) regimen could improve the outcome of patients with hematological malignancies since 1990 in the Hokkaido University Hospital. Our preliminary study in the early 1990s showed that higher ETP doses (20 and 25 mg/kg/day for 2 days) were more toxic to the patients; therefore, we fixed the ETP dose to 15 mg/kg/day for 2 days in 1993. Since then, almost all of the patients with hematological malignancies admitted to the Hokkaido University Hospital were treated with this regimen. Ten years later, we recognized that this regimen was more suitable for ALL (n = 11, the 5-Y OS: 100 %) as compared with total cases including other hematological malignancies (n = 27, the 5-Y OS: 74 %) and that remission status was important for obtaining a better outcome. A 5-Y DFS rate in CR1, CR2, and non-CR were 91 %, 83 %, and 41 %, respectively [71].

In a retrospective analysis in which 37 ALL patients in 28 CR1, 7 CR2, and 2 non-CR were treated with allo HSCT by a medium-dose ETP (15 mg/kg × 2) + CY (60 mg/kg × 2) + TBI (2 Gy × 6) regimen, a 3-Y OS, relapse, and transplant-related mortality (TRM) rate were 89 %, 8 %, and 5 %, respectively [59]. Among 10 Ph^+^ ALL patients transplanted in CR1, all patients were alive, indicating that this regimen is markedly effective even in high-risk Ph^+^ ALL patients. Six out of the ten patients who were polymerase chain reaction-positive for the *bcr-*gene rearrangement at transplantation became MRD-negative after allo HSCT. Four other patients were MRD-negative at transplantation. No patients received TKIs before and after allo HSCT.

Then, we analyzed 515 patients aged 15–59 years who received allo HSCT in CR1 or CR2 with CY (60 mg/kg × 2) + TBI (12 Gy) or ETP (15 mg/kg × 2) + CY (60 mg/kg × 2) + TBI (12 Gy) regimens between 1993 and 2007 by collecting the clinical outcomes from the Japan Society for Hematopoietic Cell Transplantation (JSHCT) and the Japan Marrow Donor Program (JMDP) data bases. A 5-Y OS rate in CY + TBI and ETP + CY + TBI regimens were 55 % and 82 % (*p* = 0.003), respectively [61], suggesting that ETP + CY + TBI regimen appears to be more beneficial to adult ALL patients as compared with CY + TBI regimen.

Another ETP (40 mg/kg in 8 h: 4 consecutive infusions of 10 mg/kg lasting 2 h each) + CY + TBI regimen was reported by a French group [25]. A 6-Y OS rate in 39 ALL patients aged < 50 years or 15–60 years, who received allo BMT in CR1 was 75 % and 41 %, respectively, whereas a 6-Y OS rate was 40 % in those who received late high-dose chemotherapy followed by auto HSCT.

Major difference between ours and theirs are as follows: we used an ETP dose of 15 mg/kg/day × 2 days and they used 40 mg/kg/day × 1 day. Furthermore, they used bone marrow (BM) stem cells alone; by contrast, among 37 patients, we used BM in 86 % of the transplants, peripheral blood (PB) stem cells in 11 %, and cord blood (CB) stem cells in 3 %. The outcome seems to be no difference in each stem cell used. Our study had 69 % high-risk ALL patients, including 27 % Ph^+^ patients [59], whereas their study had 33 % high-risk ALL patients, including 21 % Ph^+^ patients. Although they used only an HLA-matched sibling donor, we used an HLA-matched related or unrelated donor and an HLA-mismatched related or unrelated donor.

### A prospective multi-center phase II clinical trial with the medium-dose ETP + CY + TBI regimen for adult ALL patients in Japan

A prospective multi-center phase II clinical trial has been conducted in Japan to validate whether the medium-dose ETP + CY + TBI regimen is an excellent conditioning regimen for ALL patients in CR1 and CR2. The sequential order of each agent was not always fixed depending on the physician’s discretion. Of the 50 patients (47 CR1 and 3 CR2), 84 % were high-risk patients, including 38 % Ph^+^ patients. A 1-Y OS, EFS, relapse, and NRM rate were 80 %, 76 %, 10 %, and 14 %, respectively [60]. Furthermore, an estimated 2-Y OS, EFS, relapse, and NRM rate were 67 %, 65 %, 15 %, and 20 %. The relapse rate in the recent study was slightly higher than the previous rate (10 % at 1 year and 15 % at 2 years versus 8 % at 3 years); it was 10 % at 6 years in the French group study [25]. Although the 1- and 2-Y NRM rates (14 % and 20 %) in the recent study were higher than the 3-Y TRM rate (6 %) in the previous one, these rate was comparable with that of the French group study (15 %) [25]. Therefore, these results strongly confirm that this regimen is remarkably effective for adult ALL patients in CR1, showing a higher OS rate without increasing TRM/NRM and relapse rates. The outcome was not influenced by the difference of donor type (related versus unrelated), age difference (≧40 years old versus < 40 years old), stem cell source (BM versus PB), Ph-positivity (positive versus negative), risk group (high versus standard), or disease status (CR1 versus CR2). TKIs were used in Ph^+^ ALL patients only before allo HSCT. All patients were engrafted with median neutrophil recovery (≧500/μl) at day 16, similar to the result of the previous report [59]. No patients died within 100 days.

Acute and chronic graft-versus-host disease (GVHD) generally developed at rates of 20–40 % (grade ≧II: approximately 20 %) and 30–50 % (extensive type: approximately 30 %) in MAC regimens, respectively [2, 6, 7, 57, 59–61, 64]. The occurrence of acute GVHD in the medium-dose ETP + CY + TBI regimen appears to be more frequent (66 %). Although grade ≧II acute GVHD were observed in 58 % of transplants, uncontrollable severe acute GVHD was not observed. Chronic GVHD was observed in 56 % of patients (extensive type: 41 %), showing a slightly more frequent incidence. In Japan, we use both related and unrelated HLA-matched donors (approximately 35 % and 50 %, respectively), whereas HLA-matched sibling donors were generally selected in other ETP-based regimens in Western countries. The outcome of allo HSCT from an HLA-matched unrelated donor is almost the same as that from an HLA-matched related donor in Japan [26]. This favorable phenomenon may be due to Japanese genetic homogeneity [43, 44, 48, 55]. Furthermore, although we use HLA-mismatched related donors in approximately 5 % of transplants and HLA-mismatched unrelated donors in approximately 10 % of transplants, no unfavorable outcome was observed.

The upper age limit for allo HSCT is thought to be 50–55 years, although we performed allo HSCT in an ALL patient aged 58 years using the medium-dose ETP + CY + TBI regimen. When the patient was older, severe complications developed more often. Therefore, the medium-dose ETP + CY + TBI regimen appears to be appropriate for patients aged < 50 years because of its toxicity. In fact, the outcomes of RIC and MAC in allo HSCT for adult ALL patients aged ≧45 years were analyzed in the adult ALL Working Group of the JSHCT, suggesting that patients aged ≧50 years should be transplanted by RIC regimens [69].

A TRM rate of only 5 % by the medium-dose ETP + CY + TBI regimen was observed in ALL patients aged 15–58 years (54 % were from < 40-years-old patients) in our previous study [59], and a NRM rate of 14 % was observed in ALL patients aged 17–49 years in our recent study [60]. Such low TRM/NRM rates in adult ALL patients are remarkable when compared with other ETP-based regimens, in which higher doses of ETP were used. Possible explanations for this difference appears to be due to the divided administration of a lower dose of ETP and relatively younger patients enrolled (54 % were < 45-years-old). Even if lower doses of ETP were used, the relapse rate remained < 10 %. Furthermore, no patients developed second malignancies after a median follow-up period of 90 months as of June, 2014. This finding is supported by the fact that the cumulative risk factor after 15 years was 1.26 % in 366 out of 1376 ALL patients who received allo HSCT with fractionated TBI + ETP 40–60 mg/kg [8].

Although CB was not used in the present phase II trial, a similar outcome between CB and other stem cells is expected with the medium-dose ETP + CY + TBI regimen as shown in several studies [20, 31, 52, 68].

ETP-based conditioning regimens were also used in child and adolescent ALL patients [19, 72]. The ETP doses were comparable with our regimen; however, most of the patients were transplanted at advanced stages and ETP was administered in a single dose, resulting in higher TRM rates.

### Outcome of Ph^+^ adult ALL patients transplanted with allogeneic HSCT

Although some Ph^+^ ALL patients were included in each trial shown in Table 1, the outcome of allogeneic HSCT for only Ph^+^ ALL patients is shown in Table 2 [4, 17, 21, 22, 29, 62, 65, 75]. Ph^+^ALL patients who received allo HSCT after conditioning regimens excluding ETP had a poor prognosis. ETP (higher doses: 50–60 mg/kg)-based conditioning regimen improved the outcome to some extent. However, the relapse and TRM/NRM rates remained high.

**Table 2:** Myeloablative conditioning regimens in allogeneic hematopoietic stem cell transplantation for Philadelphia chromosome-positive acute lymphoblastic leukemia

Regimen	No. of ALL	Mean age (range)	Donor	Stem cell source	Disease status at HSCT	Survival rate	Relapse rate	TRM/NRM	aGVHD	cGVHD	Remarks	Reference
ETP 60 mg/kg × 1 + TBI 1.2 Gy × 11	5	28 (23–45)	MRD	BM	CR1: 4	OS: 60 % (≧171 d)	0 %	40 %	50 % (gr.≧II: 33 %)	ND	Intensified conditioning regimens followed by allogeneic HSCT were a preferred treatment modality for Ph + ALL according to a retrospective sutdy.	Forman SJ, et al. Blood 1987; 70:587
ETP 60 mg/kg × 1 + TBI 7.5 Gy × 1	1				Non-CR: 6							
CY 100 mg/kg × 1 + TBI 1.2 Gy × 11	3											
BU 1 mg/kg × 16 + CY 60 mg/kg × 1	1											
BU + CY +TBI	67	28 (5–49)	MRD	BM	CR1: 33	2-Y DFS: 38 %	34 %	CR1: 42 %	Gr.≧II: 24 %	33 %	Allogeneic HSCT was feasible for Ph^+^ ALL patients according to a retrospective study using IBMTR data. The dose of conditioning regimens was not specified.	Barrett AJ, et al. Blood 1992; 79:3067.
					Rel: 22	2-Y DFS: 41 %	32 %	Rel: 40 %	: 38 %	31 %		
					IF: 12	2-Y DFS: 25 %	57 %	IF : 42 %	: 45 %	22 %		
CY 60 mg/kg × 2 + TBI 1.2 Gy × 11 (for patients ≧18 y)	15	25 (17–51)	MUD	BM	CR1: 7	2-Y DFS: 49 %	29 %	22 %	100 % (gr.≧II: 75 %)	62 % (ext. 54 %)	Unrelated HLA-matched donors were useful when related HLA-matched donors were not found in a retrospective study.	Sierra J, et al. Blood 1997; 90:1410.
CY 60 mg/kg × 2 + TBI 1.2 Gy × 12 (for children)	3				>CR1: 1							
					Non-CR: 10							
ETP 50 mg/kg × 1 + TBI 1.2 Gy × 11+/–CY 60 mg/kg x 2	22	30 (6–42)	MRD	BM/PB	CR1	3-Y DFS: all 65 %	12 %	30 %	65 %	65 %	The relatively low relapse rate might reflect the enhanced anti-leukemic activity of ETP/TBI compared to other conditioning regimens in a retrospective study.	Snyder DS, et al. Leukemia 1999; 13:2053.
CY 60 mg/kg × 2 + TBI 1.2 Gy × 11	1					<1992 : 45 %						
						≧1992: 81 %						
ETP 50 mg/kg × 1 +CY 60 mg/kg × 2 +TBI 2 Gy × 6 or TBI 10 Gy × 1	74	42 (17–56)	MRD: 43	BM	CR1	2-Y OS: 37 %	40 %	67 %	ND	ND	Allogeneic HSCT in CR1 was the best treatment option in Ph^+^ ALL according to a prospective study. Outcome was better in bcr/abl transcript-negative patients than in bcr-abl transcript-positive ones. The 2-Y OS, relapse, and TRM rates were 26 %, 74 %, and 91 %, respectively in 23 cases of autologous HSCT.	Dombret H, et al. Blood 2002;100:2357.
			MUD: 8			2-Y OS: 63 %	25 %	38 %	ND	ND		
CY 60 m/kg × 2 + TBI 2Gy × 6 (for adults)	Adults: 102	35 (1–53)	MRD: 79	BM/PB	CR1: 76	2-Y OS: all 37 %	44 %	38 %	Adults Gr.≧II: 52 %	43 % (ext.: 17 %)	A better outcome was correlated with remission status at transplant in a prospective study. The relapse rate decreased with the occurrence of acute GVHD. Bcr/abl transcript-positivity did not correlate to relapse rate.	Espérou H, et al. Bone Marrow Transplant. 2003;31:909.
Ara-C 3 g/m^2^ × 4 or 6 + Mel 140 mg/m^2^ × 1 +TBI 2 Gy × 6 (for children)	Children: 19		MUD: 42		>CR1: 45	CR1: 50 %	37 %		Children Gr.≧II: 53 %	39 % (ext.: 8 %)		
CY 60 mg/kg × 2 + TBI 2 Gy × 6	166	37 (16–59)	MRD: 136	BM: 167	CR1: 93	5-Y OS	N.D.	N.D.	N.D.	N.D.	Allogeneic HSCT was the only procedure with curative potential for Ph^+^ ALL according to a retrospective study. Pre-transplant disease status was an important factor for better survival. Extensive cGVHD correlated with a better outcome, while severe aGVHD did not.	Yanada M. et al. Bone Marrow Transplant. 2005;36:867.
Non-TBI	31		MUD: 61	PB: 24	:>CR1 19	CR1: 34 %						
				BM + PB: 6	Non-CR: 85	>CR1: 21 %						
						5-Y OS						
						TBI (n = 166): 25 %						
						Non-TBI (n = 31): 8 %						
ETP 60 mg/kg × 1 +TBI 1.2 Gy × 11	67	36 (2–57)	MRD	BM: 43	CR1:49	10-Y OS		CR1: 31 %	49 % (gr.≧II: 35 %)	38 % (ext.: 13 %)	Disease status at the time of HSCT was important according to a retrospective study. ETP + TBI with or without CY conferred long-term survival. Seventeen patients received imatinib before HSCT and received the drug after HSCT as well.	Laport GG, et al.: Blood 2008;112:903.
ETP 60 mg/kg × 1 + CY 60 mg/kg × 2 + TBI 1.2 Gy × 11	11			PB: 36	>CR1:30	CR1: 48 %	CR1: 28 %	>CR1: 54 %				
ETP 60 mg/kg × 1+BU 1 mg/kg × 16 +TBI 1.2 Gy × 11	1					>CR1: 29 %	>CR1: 41 %					

*No.* the number of patients, *ALL* acute lymphoblastic leukemia, *HSCT* hematopoietic stem cell transplantation, *TRM* transplant-related mortality, *NRM* non-relapse mortality, *aGVHD* acute graft-versus-host disease, *cGVHD* chronic graft-versus-host disease, *ETP* etoposide, *TBI* total body irradiation, *CY* cyclophosphamide, *BU* busulfan, *MRD* matched related donor, *BM* bone marrow, *CR* complete remission, *OS* overall survival, *d* day, *gr.* grade, *ND* not determined, *Rel* relapse, *IF* induction failure, *DFS* disease-free survival, *ext.* extensive type of chronic GVHD, *IBMTR* international bone marrow transplant registry, *MUD* matched unrelated donor, *HLA* human leukocyte antigen, *PB* peripheral blood, *Ara-C* cytosine arabinoside

On the contrary, the introduction of TKIs in combination with chemotherapies has drastically changed the outcomes of Ph^+^ALL patients, resulting in increased CR rate compared with historical controls. However, since the probability of relapse remains high, allo HSCT in CR1 is required to obtain a better outcome. There are several reports regarding the efficacy of imatinib (IM) use before allo HSCT with 2-Y - 5-Y OS of 40–78 % [28, 33, 38, 50, 74].

In terms of IM use in ALL patients after allo HSCT, a 5-Y DFS in prophylactic arm and in MRD-triggered arm was 84 % and 60 % (*p* = 0.89), respecticvely and a 5-Y OS was 80 % and 75 % (*p* = 0.84), respectively [50]. Although prophylactic use of IM significantly reduced the incidence of molecular recurrence after allo HSCT compared with MRD-triggered use of IM (40 % versus 69 %, *p* = 0.046), the outcome was shown to be no difference between both arms. Relapse probability was significantly higher in patients who became MRD-positive (*p* = 0.017).

By contrast, neither TKI use pre- nor post-allo HSCT was found to significantly impact outcomes [28]. However, this is a retrospective study and there are some limitations regarding the heterogeneity in conditioning regimens, allotype, and stem cell source, as well as the small sample size.

Therefore, the outcome of chemotherapy combined with TKIs followed by allo HSCT in CR1 with MAC regimens other than ETP-based regimens appears to be not always superior to that of allo HSCT performed by using the medium-dose ETP + CY + TBI regimen. It is meaningful to evaluate whether administration of other TKIs pre- or post-allo HSCT is beneficial for the outcome of Ph^+^ ALL patients by the prospective clinical trial in the future.

### Importance of administration schedule of ETP

The administration schedule of ETP is an important factor for reducing adverse effects, although it depends on combined chemotherapy and/or TBI. We used a 3-h infusion on 2 consecutive days with 30 mg/kg of ETP, whereas 60 mg/kg of ETP was usually administered in a single dose of a 4-h infusion [2, 6, 7, 64]. The maximally tolerated dose (MTD) is thought to be 2.4 g/m^2^ in 1 to 1.5-h infusions on 3 consecutive days, with 4.2 g/m^2^ as continuous infusion (29 to 69-h) [9, 24]. When ETP, CY, and TBI were combined, the MTDs were 1.8 g/m^2^ for ETP, and 180 mg/kg for CY.

### Possible mechanisms of efficacy of the medium-dose ETP + CY + TBI

It is important to clarify how the medium-dose ETP + CY + TBI regimen can eradicate residual leukemic cells more efficiently than the CY + TBI regimen or others. The graft-versus-leukemia (GVL) effect is hardly observed against ALL; however, allo HSCT is superior to auto HSCT in terms of the outcome [18, 23, 70]. Furthermore, chronic GVHD appears to induce a GVL effect in ALL patients [32], although others could not confirm a decreased relapse risk in the higher incidence of chronic GVHD among the recipients of allo PB [10, 56].

ETP upregulated the expression levels of interleukin (IL)-8 and macrophage inflammatory protein-1α in promonocytic acute myelogenous leukemia cells, most likely inducing an enhancement of the anti-leukemic effect [45]. A low dose of ETP can enhance leukemia rejection in mice by rendering them immunogenic and susceptible to CD8^+^T cell deaths [63]. Several immunomodulations by ETP are also observed in other tumor cells [73]. A preliminary *in vitro* study showed that CY synergized with ETP in terms of cytotoxic effect against leukemic cells (unpublished observation). Indeed, it is known that ETP exhibits a synergy with 4-hydroperxycyclophosphamide against a promyelocytic leukemic cell line determined by *in vitro* cytotoxicity assay [11]. Therefore, some of these mechanisms may be responsible for the superiority of the medium-dose ETP + CY + TBI regimen in ALL patients.

### Reduced-intensity conditioning regimens for adult ALL patients

As shown in Table 3, various RIC regimens for allo HSCT were applied for adult ALL patients [3, 13, 35, 36, 41, 67]. The stem cell source was mainly either BM or PB from an HLA-matched or mismatched related and an HLA-matched or mismatched unrelated donor. The 2-Y or 3-Y OS were 30-60 %. A better outcome was observed in patients transplanted in CR1 compared with those transplanted in more advanced stages. The relapse rates were 20–60 %. The TRM/NRM rates were 20–40 %. The occurrence of acute GVHD (≧grade II) and chronic GVHD was 40–60 % and 30–70 % (extensive type: 30–50 %), respectively. GVL effect against ALL could be induced by RIC regimens and allo HSCT with RIC regimens was feasible for patients with high-risk ALL patients in remission [36]. It is notable that the relapse rate in the RIC regimen for adult Ph^−^ ALL patients in Japan was higher (26 % vs 10 %) than that in the medium-dose ETP + CY + TBI regimen [46, 60].

**Table 3:** Reduced-intensity conditioning regimens in allogeneic hematopoietic stem cell transplantation for acute lymphoblastic leukemia

Regimen	No. of ALL	Mean age (range)	Donor	Stem cell source	Disease status at HSCT	Survival rate	Relapse rate	TRM/NRM	aGVHD	cGVHD	Remarks	Reference
Flu 90–150 mg/m^2^ + Mel 140 mg/m^2^	27	50 (18–63)	MRD	BM	CR1: 3	2-Y OS: 31 %	49 %	TRM: 23 %	Gr.≧II: 48 %	67 %	A small sample sized retrospective study; GVL effect was exhibited.	Martino R, et al. Haematologica 2003;88: 555.
Flu + Mel+ Ara-C 2 g/m^2^			MMRD	PB	CR2/3:10		GVHD+: 35 %			Limt.: 39 %		
Flu + 2 Gy TBI			MUD		PR2:1		GVHD-: 70 %			Ext.: 28 %		
Flu + Mel+ CAMPATH-1H 200 mg/kg			MMUD		Non-CR: 12							
Flu + CY 60 mg/kg+ Thiotpa 10 mg/kg												
Flu 30 mg/m^2^ × 6 +4 Gy TBI/ATG 10 mg/kg/d × 4	97	38 (15–66)	MRD	BM	CR1:28	2-Y OS: 52 %	40 %	CR1, NRM: 18 %	Gr.≧II: 33 %	37 %	Factors for better OS were CR1, chronic GVHD, and female donor according to a retrospective study.	Mohty M, et al. Haematologica 2008; 93: 303.
Flu + BU 8 mg/kg			MUD	PB	CR2/3: 30	27 %	63 %	CR2/CR3, NRM: 17 %				
Flu + Mel					Non-CR: 39	20 %	49 %	More advanced stage, NRM: 44 %				
Flu + CY												
Flu 25 mg/m^2^ × 5+ Mel 140 mg/m^2^ × 1	24	48 (23–68)	MRD	PB	CR1: 11	2-Y OS: 62 %	21 %	NRM: 22 %	Gr.≧II: 63 %	75 %	RIC HSCT might offer a promising option for high risk ALL patients not eligible for standard myeloablative transplantation according to a. retrospective study.	Stein AS, et al. Biol. Blood Marrow Transplant. 2009; 15: 1407.
			MUD		CR2: 5					Limit.: 21 %		
					≧CR3: 3					Ext.: 54 %		
					Non-CR 5							
Flu 40 mg/m^2^ × 5+ CY 50 mg/kg+ TBI 2 Gy	22 (high risk)	49 (24–68)	MRD	PB	CR1: 12	3-Y OS: 50 %	36 %	TRM: 27 %	Gr.≧II:55 %	45 %	In a small sample sized prospective study, HSCT at CR1 showed an excellent outcome, but the relapse rate was high.	Bachanova V, et al. Blood 2009; 113: 2902.
			MUD	CB	≧CR2: 10				Gr.≧III: 20 %	Ext.: 32 %		
Flu 30 mg/m^2^ × 5 +Mel 70 mg/m^2^ × 2 +/−ATG 2.5 mg/kg	37	45 (15–63)	MRD	PB	CR1: 30	3-Y OS: 64 %	20 %	NRM: 18 %	Gr.≧II: 43 %	66 %	Transplant in CR1 showed a better outcome in a prospective phase II study. GVL effect was induced in cGVHD.	Cho B-S, et al. Leukemia 2009;23:1763.
			MUD	BM	CR2: 7					Limit: 28 %		
			MMUD							Ext: 38 %		
BU 9 mg/kg or less + Mel 150 mg/m^2^ or less+ TBI >5 Gy or fractionated	93	45 (17–66)	MRD: 30	PB: 68	CR1: 55	3-Y OS: 38 % (RIC)	35 % (RIC)	RIC			TRM rate in RIC was almost the same as that in MAC, but the relapse rate was higher in RIC (35 % vs 26 %) according to a retrospective study.	Marks DI, et al. Blood 2010;116:366
TBI >8 Gy			MUD: 36	BM: 25	CR2: 38	3-Y OS: 43 % (MAC)	26 % (MAC)	TRM: 32 %	Gr.≧II: 39 %	34 %		
Flu + TBI 2 Gy			PMMUD: 20			3-Y DFS: 32 % (RIC)		MAC				
Others			MMUD: 5			3-Y DFS: 41 % (MAC)		TRM: 33 %	Gr.≧II: 46 %	42 %		
			UD: 2									

*No.* the number of patients, *ALL* acute lymphoblastic leukemia, *HSCT* hematopoietic stem cell transplantation, *TRM* transplant-related mortality, *NRM* nonrelapse mortality, *aGVHD* acute graft-versus-host disease, *cGVHD* chronic graft-versus-host disease, *Flu* fludarabine, *Mel* melphalan, *Ara-C* cytosine arabinoside, *Gy* gray, *TBI* total body irradiation, *CY* cyclophosphamide, *MRD* matched related donor, *MMRD* mismatched related donor, *MUD* matched unrelated donor, *MMUD* mismatched unrelated donor, *BM* bone marrow, *PB* peripheral blood, *CR* complete remission, *PR* partial remission, *Y* year, *OS* overall survival, *gr.* grade, *Limit.* limited type of cGVHD, *Ext.* extensive type of cGVHD, *GVL* graft-versus-leukemia, *ATG* anti-thymocyte globulin, *BU* busulfan, *Ph* Philadelphia chromosome, *RIC* reduced-intensity conditioning, *CB* cord blood, *PMMUD* partially mismatched unrelated donor, *UD* unknown donor, *DFS* disease-free survival, *MAC* myeloablative conditioning regimen

### Pediatric-inspired regimens for adult Ph^−^ ALL patients

Pediatric-inspired regimens resulted in a better outcome for adult Ph^−^ ALL patients aged 15–55 years regardless of undergoing allo HSCT in CR1 [16]. Although CY + TBI conditioning regimen was used for allo HSCT, a 3-Y relapse rate, NRM, and relapse-free survival (RFS) were 20 %, 16 %, and 65 %, respectively. Of the allo HSCT patients, a 3 Y-NRM in the patients aged 45–55 years and those aged 15–44 years was 26 % and 13 % (*p* < 0.048), respectively. A 3-Y RFS in the younger and the older was 67 % and 56 % (*p* < 0.12), respectively and a 3-Y OS was 72 % and 61 % (*p* < 0.082), respectively.

Almost comparable outcomes were observed in patients with allo HSCT and those without allo HSCT, although the survival in patients with MRD (≧1^−3^) at 6 weeks after induction initiation was longer in the allo HSCT cohort than in the no HSCT cohort. These results indicate standard risk ALL patients aged < 40–45 years with MRD (<1^−3^) at 6 weeks after induction initiation may not require allo HSCT in CR1. A similar result has been also shown in other study [15]. In the future, it should be determined whether the medium-dose ETP + CY + TBI regimen is suitable for adult standard risk ALL patients aged < 40–45 years based on MRD analysis.

### HLA-haploidentical hematopoietic stem cell transplantation for adult ALL patients

Recently, haplo HSCT, which is varied in the settings, has been increasingly performed [51]. Basically, this procedure is still alternative for the patients lacking an HLA-matched related donor, since the outcomes are approximately 20–50 % and limited numbers of ALL cases are reported. In patients with ALL, AML, and other hematological malignancies transplanted with mostly T cell-depleted PB, DFS, relapse rate, and TRM rate were 20–50 %, 20–60 %, and 30–40 %, respectively. The outcomes in ALL patients were generally inferior to those in AML patients [1].

Among several haplo HSCT, the following method appears to be excellent for ALL patients. Adult Ph^−^ ALL patients (n = 183) in CR1 received unmanipulated haplo HSCT including granulocyte colony stimulating factor (G-CSF) for all donors, intensive immune suppression, anti-thymocyte globulin, and combination of G-CSF-primed BM and G-CSF-mobilized PB. A 3-Y relapse mortality and NRM for high risk versus low risk groups were 7 % versus 11 % (*p* = 0.498) and 18 % versus 16 % (*p* = 0.717), respectively. A 3-Y DFS and OS for high risk and low risk were 67 % versus 68 % (*p* = 0.896) and 75 % versus 73 % (*p* = 0.981), respectively [40].

The same group reported that the outcome of haplo HSCT for Ph^+^ ALL patients (n = 101) is comparable to that of HSCT (n = 38) from an HLA-matched related donor. A 5-Y DFS, OS, relapse, and NRM rates in the haplo HSCT group were 66 %, 74 %, 20 %, and 16 %, respectively. Acute (grade ≧II) GVHD was higher (32 % versus 16 %, =0.045) in the halpo HSCT than in the control group, although there was no difference between both groups in chronic GVHD frequency (extensive type: 20 % versus 13 %) [12].

Ruggeri et al. [54] reported that no statistically significant differences were observed between haplo HSCT and CB transplantation for a 5-Y relapse (approximately 40 %), NRM (approximately 30 %), and LFS (approximately 30 %). The outcomes were not so excellent probably due to the inclusion of many patients not in CR1. However, this strategy is valid for ALL patients lacking an HLA-matched donor.

## Conclusions

In allo HSCT, the medium-dose ETP + CY + TBI regimen is promising for high-risk ALL patients aged < 50 years in CR1 and also for standard-risk patients in CR2, resulting in an excellent outcome without higher TRM and relapse rates. However, the enrolled case for the prospective multi-center phase II clinical trial with the medium-dose ETP + CY + TBI regimen for adult ALL patients in Japan is still small and a prospective randomized phase III clinical trial is required for determining its genuine efficacy in the future. On the contrary, RIC for adult ALL patients is preferable to older patients aged ≧50 years or younger patients with serious comorbid conditions and who are MRD-negative. Furthermore, recently developed various therapeutic strategies such as haplo-HSCT, pediatric-inspired regimens, and use of TKIs at pre- or post-allo HSCT for adult ALL patients should be taken into consideration to obtain a better clinical outcome (Table 4).

**Table 4:** Possible treatments for adult ALL patients by Ph-positivity, age, and MRD-positivity

Ph	Age (years)	Induction therapy	MRD	Allo HSCT
−	<45	Pediatric-inspired Chemotherapy	+	Allo HSCT with medium-dose ETP + CY + TBI in CR1/2 (Haplo HSCT with MAC in the case of lacking an HLA-matched donor in CR1)
				No HSCT
	45–50	Conventional chemotherapy		Allo HSCT with medium-dose ETP + CY + TBI in CR1/2 (Haplo HSCT with MAC in the case of lacking an HLA-matched donor in CR1)
	≧50	Conventional chemotherapy	−	Allo HSCT with RIC in CR1 (Haplo HSCT with RIC in the case of lacking an HLA- matched donor in CR1)
+	<45	Conventional or pediatric-inspired chemotherapy + TKI		Allo HSCT with medium-dose ETP + CY + TBI in CR1 (Haplo HSCT with MAC in the case of lacking an HLA-matched donor in CR1)
	45–50	Conventional chemotherapy + TKI		Allo HSCT with medium-dose ETP + CY + TBI in CR1/2 (Haplo HSCT with MAC in the case of lacking an HLA-matched donor in CR1)
	≧50	Conventional chemotherapy + TKI	−	Allo HSCT with RIC in CR1 (Haplo HSCT with RIC in the case of lacking an HLA-matched donor in CR1)

*ALL* acute lymphoblastic leukemia, *Ph* Philadelphia chromosome, *MRD* minimal residual disease, *Allo* allogeneic, *ETP* etoposide, *CY* cyclophosphamide, *TBI* total body irradiation, *CR* complete remission, *HLA* human leukocyte antigen, *Haplo HSCT* HLA-haploidentical hematopoietic stem cell transplantation, *MAC* myeloablative conditioning, *RIC* reduced-intensity conditioning, *TKI* tyrosine kinase inhibitor

## References

[1] Aversa F (2008). Haploidentical haematopoietic stem cell transplantation for acute leukaemia in adults: experience in Europe and the United States. Bone Marrow Transplant.

[2] Bachanova V, Marks DI, Zhang M-J, Wang H, De Lima M, Aljurf MD (2014). Ph + ALL patients in first complete remission have similar survival after reduced intensity and myeloablative allogeneic transplantation: impact of tyrosine kinase inhibitor and minimal residual disease. Leukemia.

[3] Bachanova V, Verneris MR, DeFor T, Brunstein CG, Weisdorf DJ (2009). Prolonged survival in adults with acute lymphoblastic leukemia after reduced-intensity conditioning with cord blood or sibling donor transplantation. Blood.

[4] Barrett AJ, Horowitz MM, Ash RC, Atkinson K, Gale RP, Goldman JM (1992). Bone marrow transplantation for Philadelphia chromosome-positive acute lymphoblastic leukemia. Blood.

[5] Bishop MR, Logan BR, Gandham S, Bolwell BJ, Cahn JY, Lazarus HM (2008). Long-term outcomes of adults with acute lymphoblastic leukemia after autologous or unrelated donor bone marrow transplantation: a comparative analysis by the National Marrow Donor Program and Center for International Blood and Marrow Transplant Research. Bone Marrow Transplant.

[6] Blume KG, Forman SJ, O’Donnell MR, Doroshow JH, Krance RA, Nademanee AP (1987). Total body irradiation and high-dose etoposide: a new preparatory regimen for bone marrow transplantation in patients with advanced hematologic malignancies. Blood.

[7] Blume KG, Kopecky KJ, Henslee-Downey JP, Forman SJ, Stiff PJ, LeMaistre CF (1993). A prospective randomized comparison of total body irradiation-etoposide versus busulfan-cyclophosphamide as preparative regimens for bone marrow transplantation in patients with leukemia who were not in first remission: a Southwest Oncology Group Study. Blood.

[8] Borgmann A, Zinn C, Hartmann R, Herold R, Kaatsch P, Escherich G (2005). Secondary malignant neoplasms after intensive treatment of relapsed acute lymphoblastic leukaemia in childhood. Eur J Cancer.

[9] Brown RA, Herzig RH, Wolff SN, Frei-Lahr D, Pineiro L, Bolwell RA (1990). High-dose etoposide and cyclophosphamide without bone marrow transplantation for resistant hematologic malignancy. Blood.

[10] Champlin RE, Schmitz N, Horowitz MM, Chapuis B, Chopra R, Cornelissen JJ (2000). Blood stem cells compared with bone marrow as a source of hematopoietic cells for allogeneic transplantation: IBMTR Histocompatibility and Stem Cell Sources Working Committee and the European Group for Blood and Marrow Transplantation (EBMT). Blood.

[11] Chang T, Gulati S, Chou T, Vega R, Gandola L, Ibrahim SM (1985). Synergistic effect of 4-hydroperoxycyclophosphamide and etoposide on a human promyelocytic leukemia cells line (HL-60) demonstrated by computer analysis. Cancer Res.

[12] Chen H, Liu KY, Xu LP, Chen YH, Han W, Zhang XH (2015). Haploidentical hematopoietic stem cell transplantation without in vitro T cell depletion for the treatment of Philadelphia chromosome-positive acute lymphoblastic leukemia. Biol Blood Marrow Transplant.

[13] Cho BS, Lee S, Kim YJ, Chung NG, Eom KS, Kim HJ (2009). Reduced-intensity conditioning allogeneic stem cell transplantation is a potential therapeutic approach for adults with high-risk acute lymphoblastic leukemia in remission: results of a prospective phase 2 study. Leukemia.

[14] Cornelissen JJ, van der Holt B, Verhoef GE, van’t Veer MB, Van Oers MH, Schouten HC (2009). Myeloablative allogeneic versus autologous stem cell transplantation in adult patients with acute lymphoblastic leukemia in first remission: a prospective sibling donor versus no-donor comparison. Blood.

[15] DeAngelo DJ, Stevenson KE, Dahlbery SE, Silverman LB, Couban S, Supko JG (2015). Long-term outcome of a pediatric-inspired regimen used for adults aged 18–50 years with newly diagnosed acute lymphoblastic leukemia. Leukemia.

[16] Dhédin N, Huynh A, Maury S, Tabrizi R, Beldjord K, Asnafi V (2015). Role of allogeneic stem cell transplantation in adult patients with Ph-negative acute lymphoblastic leukemia. Blood.

[17] Dombret H, Gabert J, Boiron JM, Rigal-Huguet F, Blaise D, Thomas X (2002). Outcome of treatment in adults with Philadelphia chromosome-positive acute lymphoblastic leukemia: results of the prospective multicenter LALA-94 trial. Blood.

[18] Doney K, Hägglund H, Leisenring W, Chauncey T, Appelbaum FR, Storb R (2003). Predictive factors for outcome of allogeneic hematopoietic cell transplantation for adult acute lymphoblastic leukemia. Biol Blood Marrow Transplant.

[19] Duerst RE, Horan JT, Liesveld JL, Abboud CN, Zwetsch LM, Senf ES (2000). Allogeneic bone marrow transplantation for children with acute leukemia: cytoreduction with fractionated total body irradiation, high-dose etoposide and cyclophosphamide. Bone Marrow Transplant.

[20] Eapen M, Rocha V, Sanz G, Scaradavou A, Zhang MJ, Arcese W (2010). Effect of graft source on unrelated donor haemopoietic stem-cell transplantation in adults with acute leukaemia: a retrospective analysis. Lancet Oncol.

[21] Espérou H, Boiron JM, Cayuela JM, Blanchet O, Kuentz M, Jouet J-P (2003). A potential graft-versus-leukemia effect after allogeneic hematopoietic stem cell transplantation for patients with Philadelphia chromosome-positive acute lymphoblastic leukemia: results from the French Bone Marrow Transplantation Society. Bone Marrow Transplant.

[22] Forman SJ, O’Donnell MR, Nademanee AP, Snyder DS, Bierman PJ, Schmidt GM (1987). Bone marrow transplantation for patients with Philadelphia chromosome-positive acute lymphoblastic leukemia. Blood.

[23] Goldstone AH, Richards SM, Lazarus HM, Tallman MS, Buck G, Fielding AK (2008). In adults with standard-risk acute lymphoblastic leukemia, the greatest benefit is achieved from a matched sibling allogeneic transplantation in first complete remission, and an autologous transplantation is less effective than conventional consolidation/maintenance chemotherapy in ALL patients: final results of the International ALL trial (MRC UKALL XII/ECOG E2993). Blood.

[24] Herzig RH (1991). High-dose etoposide and marrow transplantation. Cancer.

[25] Hunault M, Harousseau JL, Delain M, Truchan-Graczyk M, Cahn JY, Witz F (2004). Better outcome of adult acute lymphoblastic leukemia after early genoidentical allogeneic bone marrow transplantation (BMT) than after late high-dose therapy and autologous BMT: a GOELAMS trial. Blood.

[26] Imamura M, Asano S, Harada M, Ikeda Y, Kato K, Kato S (2006). Current status of hematopoietic cell transplantation for adult patients with hematological diseases and solid tumors. Int J Hematol.

[27] Jamieson CHM, Amylon MD, Wong RM, Blume KG (2003). Allogeneic hematopoietic cell transplantation for patients with high-risk acute lymphoblastic leukemia in first or second remission using fractionated total-body irradiation and high-dose etoposide: A 15-year experience. Exp Hematol.

[28] Kebriaei P, Saliba R, Rondon G, Chiattone A, Luthra R, Anderlini P (2012). Long-term follow-up of allogeneic hematopoietic stem cell transplantation for patients with Philadelphia chromosome positive acute lymphoblastic leukemia: impact of tyrosine kinase inhibitors on treatment outcomes. Biol Blood Marrow Transplant.

[29] Laport GG, Alvarnas JC, Palmer JM, Snyder DS, Slovak ML, Cherry AM (2008). Long-term remission of Philadelphia chromosome-positive acute lymphoblastic leukemia after allogeneic hematopoietic cell transplantation from matched sibling donors: a 20-year experience with the fractionated total body irradiation-etoposide regimen. Blood.

[30] Larson RA, Dodge RK, Burns CP, Lee EJ, Stone RM, Schulman P (1995). A five-drug remission induction regimen with intensive consolidation for adults with acute lymphoblastic leukemia: Cancer and Leukemia Group B study 8811. Blood.

[31] Laughlin MJ, Eapen M, Rubinstein P, Wagner JE, Zhang MJ, Champlin RE (2004). Outcomes after transplantation of cord blood or bone marrow from unrelated donors in adults with leukemia. N Engl J Med.

[32] Lee S, Cho BS, Kim SY, Choi SM, Lee DG, Eom KS (2007). Allogeneic stem cell transplantation on first complete remission enhances graft-versus-leukemia effect in adults with acute lymphoblastic leukemia: antileukemic activity of chronic graft-versus-host disease. Biol Blood Marrow Transplant.

[33] Lee S, Kim YJ, Min CK, Kim HJ, Eom KS, Kim DW (2005). The effect of first-line imatinib interim therapy on the outcome of allogeneic stem cell transplantation in adults with newly diagnosed Philadelphia chromosome-positive acute lymphoblastic leukemia. Blood.

[34] Marks DI, Forman SJ, Blume KG, Pérez WS, Weisdorf DJ, Keating A (2006). A comparison of cyclophosphamide and total body irradiation with etoposide and total body irradiation as conditioning regimens for patients undergoing sibling allografting for acute lymphoblastic leukemia in first or second complete remission. Biol Blood Marrow Transplant.

[35] Marks DI, Wang T, Pérez WS, Antin JH, Copelan E, Gale RP (2010). The outcome of full-intensity and reduced-intensity conditioning matched sibling or unrelated donor transplantation in adults with Philadelphia chromosome-negative acute lymphoblastic leukemia in first and second complete remission. Blood.

[36] Martino R, Giralt S, Caballero MD, Mackinnon S, Corradini P, Fernández-Avilés F (2003). Allogeneic hematopoietic stem cell transplantation with reduced-intensity conditioning in acute lymphoblastic leukemia: a feasibility study. Haematologica.

[37] Mengarelli A, Iori AP, Guglielmi C, Romano A, Cerretti R, Torromeo C (2002). Standard versus alternative myeloablative conditioning regimens in allogeneic hematopoietic stem cell transplantation for high-risk acute leukemia. Haematologica.

[38] Mizuta S, Matsuo K, Nishiwaki S, Imai K, Kanamori H, Ohashi K (2014). Pretransplant administration of imatinib for allo-HSCT in patients with BCR-ABL-positive acute lymphoblastic leukemia. Blood.

[39] Mizuta S, Matsuo K, Yagasaki F, Yujiri T, Hatta Y, Kimura Y (2011). Pre-transplant imatinib-based therapy improves the outcome of allogeneic hematopoietic stem cell transplantation for BCR-ABL-positive acute lymphoblastic leukemia. Leukemia.

[40] Mo XD, Xu LP, Zhang XH, Liu DH, Wang Y, Chen H (2015). Haploidentical hematopoietic stem cell transplantation in adults with Philadelphia-negative acute lymphoblastic leukemia: no difference in the high- and low-risk groups. Int J Cancer.

[41] Mohty M, Labopin M, Tabrizzi R, Theorin N, Fauser AA, Rambaldi A (2008). Reduced intensity conditioning allogeneic stem cell transplantation for adult patients with acute lymphoblastic leukemia: a retrospective study from the European Group for Blood and Marrow Transplantation. Haematologica.

[42] Mohty M, Labopin M, Volin L, Gratwohl A, Socié G, Esteve J (2010). Reduced intensity versus conventional myeloablative conditioning allogeneic stem cell transplantation for patients with acute lymphoblastic leukemia: a retrospective study from the European Group for Blood and Marrow Transplantation. Blood.

[43] Morishima Y, Kawase T, Malkki M, Morishima S, Spellman S, Kashiwase K (2013). Significance of ethnicity in the risk of acute graft-versus-host disease and leukemia relapse after unrelated donor hematopoietic stem cell transplantation. Biol Blood Marrow Transplant.

[44] Morishima Y, Morishita Y, Tanimoto M, Ohno R, Saito H, Horibe K (1989). Low incidence of acute graft-versus-host disease by the administration of methotrexate and cyclosporine in Japanese leukemia patients after bone marrow transplantation from human leukocyte antigen compatible siblings: possible role of genetic homogeneity. The Nagoya Bone Marrow Transplantation Group. Blood.

[45] Mühl H, Nold M, Chang JH, Frank S, Eberhardt W, Pfeilschifter J (1999). Expression and release of chemokines associated with apoptotic cell death in human promonocytic U937 cells and peripheral blood mononuclear cells. Eur J Immunol.

[46] Nishiwaki S, Inamoto Y, Imamura M, Tsurumi H, Hatanaka K, Kawa K (2011). Reduced-intensity versus conventional myeloablative conditioning for patients with Philadelphia chromosome-negative acute lymphoblastic leukemia in complete remission. Blood.

[47] Nishiwaki S, Inamoto Y, Sakamaki H, Kurokawa M, Iida H, Ogawa H (2010). Allogeneic stem cell transplantation for adult Philadelphia chromosome-negative acute lymphocytic leukemia: comparable survival rates but different risk factors between related and unrelated transplantation in first remission. Blood.

[48] Petersdorf EW, Longton GM, Anasetti C, Martin PJ, Mickelson EM, Smith AG (1995). The significance of HLA-DRB1 matching on clinical outcome after HLA-A, B, DR identical unrelated donor marrow transplantation. Blood.

[49] Petersen FB, Buckner CD, Appelbaum FR, Sanders JE, Bensinger WI, Storb R (1992). Etoposide, cyclophosphamide and fractionated total body irradiation as a preparative regimen for marrow transplantation in patients with advanced hematological malignancies: a phase I study. Bone Marrow Transplant.

[50] Pfeifer H, Wassman B, Bethge W, Dengler J, Bornhauser M, Stadler M (2013). Randomized comparison of prophylactic and minimal residual disease-triggered imatinib after allogeneic stem cell transplantation for BCR-ABL1-positive acute lymphoblastic leukemia. Leukemia.

[51] Reisner Y, Aversa F, Martelli MF (2015). Haploidentical hematopoietic stem cell transplantation: state of art. Bone Marrow Transplant.

[52] Rocha V, Labopin M, Sanz G, Arcese W, Schwerdtfeger R, Bosi A (2004). Transplants of umbilical-cord blood or bone marrow from unrelated donors in adults with acute leukemia. N Engl J Med.

[53] Rowe JM, Buck G, Burnett AK, Chopra R, Wiernik PH, Richards SM (2005). Induction therapy for adults with acute lymphoblastic leukemia: results of more than 1500 patients from the international ALL trial: MRC UKALL XII/ECOG E2993. Blood.

[54] Ruggeri A, Labopin M, Sanz G, Piemontese S, Arcese W, Bacigalupo A (2015). Comparison of outcomes after unrelated cord blood and unmanipulated haploidemntical stem cell transplantation in adults with acute leukemia. Leukemia.

[55] Sasazuki T, Juji T, Morishima Y, Kinukawa N, Kashiwabara H, Inoko H (1998). Effect of matching of class I HLA alleles on clinical outcome after transplantation of hematopoietic stem cells from an unrelated donor. N Engl J Med.

[56] Schmitz N, Beksac M, Hasenclever D, Bacigalupo A, Ruutu T, Nagler A (2002). Transplantation of mobilized peripheral blood cells to HLA-identical siblings with standard-risk leukemia. Blood.

[57] Schmitz N, Gassmann W, Rister M, Johannson W, Suttorp M, Brix F (1988). Fractionated total body irradiation and high-dose VP16-213 followed by allogeneic bone marrow transplantation in advanced leukemias. Blood.

[58] Sebban C, Lepage E, Vernant JP, Gluckman E, Attal M, Reiffers J (1994). Allogeneic bone marrow transplantation in adult acute lymphoblastic leukemia in first remission: a comparative study. French Group of Therapy of Adult Acute Lymphoblastic Leukemia. J Clin Oncol.

[59] Shigematsu A, Kondo T, Yamamoto S, Sugita J, Onozawa M, Kahata K (2008). Excellent outcome of allogeneic hematopoietic stem cell transplantation using a conditioning regimen with medium-dose VP-16, cyclophosphamide and total-body irradiation for adult patients with acute lymphoblastic leukemia. Biol Blood Marrow Transplant.

[60] Shigematsu A, Ozawa Y, Onizuka M, Fujisawa S, Suzuki R, Atsuta Y (2015). A safety and efficacy of medium-dose etoposide, cyclophosphamide and TBI before allogeneic hematopoietic stem cell transplantation for acute lymphoblastic leukemia. Transplant Direct.

[61] Shigematsu A, Tanaka J, Suzuki R, Atsuta Y, Kawase T, Ito YM (2011). Outcome of medium-dose VP-16/CY/TBI superior to CY/TBI as a conditioning regimen for allogeneic stem cell transplantation in adult patients with acute lymphoblastic leukemia. Int J Hematol.

[62] Sierra J, Radich J, Hansen JA, Martin PJ, Petersdorf EW, Bjerke J (1997). Marrow transplants from unrelated donors for treatment of Philadelphia chromosome-positive acute lymphoblastic leukemia. Blood.

[63] Slater LM, Sweet P, Stupecky M, Reynolds JT (1995). Cyclosporin A/VP-16 produced immunity to L1210 leukemia: the participation of cytotoxic CD8 T-lymphocytes. Clin Immunol Immunopathol.

[64] Snyder DS, Chao NJ, Amylon MD, Taguchi J, Long GD, Negrin RS (1993). Fractionated total body irradiation and high-dose etoposide as a preparatory regimen for bone marrow transplantation for 99 patients with acute leukemia in first complete remission. Blood.

[65] Snyder DS, Nademanee AP, O’Donnell MR, Parker PM, Stein AS, Margolin K (1999). Long-term follow-up of 23 patients with Philadelphia chromosome-positive acute lymphoblastic leukemia treated with allogeneic bone marrow transplantation in first complete remission. Leukemia.

[66] Spitzer TR, Peters C, Ortlieb M, Tefft MC, Torrisi J, Cahill R (1994). Etoposide in combination with cyclophosphamide and total body irradiation or busulfan as conditioning for marrow transplantation in adults and children. Int J Radiat Oncol Biol Phys.

[67] Stein AS, Palmer JM, O’Donnell MR, Kogut NM, Spielberger RT, Slovak ML (2009). Reduced-intensity conditioning followed by peripheral blood stem cell transplantation for adult patients with high-risk acute lymphoblastic leukemia. Biol Blood Marrow Transplant.

[68] Takahashi S, Iseki T, Ooi J, Tomonari A, Takasugi K, Shimohakamada Y (2004). Single institute comparative analysis of unrelated bone marrow transplantation and cord blood transplantation for adult patients with hematologic malignancies. Blood.

[69] Tanaka J, Kanamori H, Nishiwaki S, Ohashi K, Taniguchi S, Eto T (2013). Reduced-intensity vs myelablative conditioning allogeneic hematopoietic SCT for patients aged over 45 years with ALL in remission: a study from the adult ALL Working Group of the Japan Society for Hematopoietic Cell Transplantation (JSHCT). Bone Marrow Transplant.

[70] Thomas X, Boiron JM, Huguet F, Dombret H, Bradstock K, Vey N (2004). Outcome of treatment in adults with acute lymphoblastic leukemia: analysis of the LALA-94 trial. J Clin Oncol.

[71] Toubai T, Tanaka J, Mori A, Hashino S, Kobayashi S, Ota S (2004). Efficacy of etoposide, cyclophosphamide, and total body irradiation in allogeneic bone marrow transplantation for adult patients with hematological malignancies. Clin Transplant.

[72] Tracey J, Zhang M-J, Thiel E, Sobocinski KA, Eapen M (2013). Transplantation conditioning regimens and outcomes after allogeneic hematopoietic cell transplantation in children and adolescents with acute lymphoblastic leukemia. Biol Blood Marrow Transplant.

[73] Wan S, Pestka S, Jubin RG, Lyu YL, Tsai YC, Liu LF (2012). Chemotheraputics and radiation stimulate MHC class I expression through elevated interferon-beta signaling in breast cancer cells. PLoS One.

[74] Wassmann B, Pfeifer H, Goekbuget N, Beelen DW, Beck J, Stelljes M (2006). Alternating versus concurrent schedules of imatinib and chemotherapy as front-line therapy for Philadelphia-positive acute lymphoblastic leukemia (Ph + ALL). Blood.

[75] Yanada M, Naoe T, Iida H, Sakamaki H, Sakura T, Kanamori H (2005). Myeloablative allogeneic hematopoietic stem cell transplantation for Philadelphia chromosome-positive acute lymphoblastic leukemia in adults: significant roles of total body irradiation and chronic graft-versus-host disease. Bone Marrow Transplant.

